# Extensive Perineural and Intraneural Invasion of a Squamous Cell Carcinoma Arising in an Epidermal Inclusion Cyst

**DOI:** 10.7759/cureus.104628

**Published:** 2026-03-03

**Authors:** Nabor S Mireles, Natalie Garcia, Isabella Camacho-Hubbard, Nisha Ramani, Jennifer S Ranario

**Affiliations:** 1 School of Medicine, Baylor College of Medicine, Houston, USA; 2 Department of Dermatology, Baylor College of Medicine, Houston, USA; 3 Department of Pathology and Immunology, Baylor College of Medicine, Houston, USA

**Keywords:** cutaneous squamous cell carcinoma, epidermal inclusion cyst, intraneural invasion, mohs micrographic surgery, perineural invasion

## Abstract

A 75-year-old man presented with a three-year history of a non-healing papule on the left eyebrow. Initial biopsy demonstrated vascular ectasia and chronic inflammation. Two years later, repeat sampling revealed poorly differentiated cutaneous squamous cell carcinoma (SCC). Mohs micrographic surgery (MMS) was subsequently performed and required seven stages. The final stage of frozen section interpretation was limited due to dense perineural inflammation. Permanent sections from the main lesion demonstrated SCC arising from the wall of an epidermal inclusion cyst (EIC) with deep dermal and subcutaneous extension, and additional en face margins submitted for permanents showed extensive perineural invasion and intraneural invasion involving multiple larger-caliber nerves. p40 immunostaining highlighted tumor nests and delineated their extension into the deep dermis and subcutaneous tissue. The patient underwent wide local excision with parotidectomy, selective neck dissection, and free-flap reconstruction, followed by adjuvant radiation. Follow-up at four months showed no recurrence. This case highlights the rarity of SCC arising within an EIC, with both extensive perineural and intraneural invasion. Frozen section interpretation was limited by dense perineural inflammation, while permanent levels and selective immunohistochemistry clarified margins and informed escalation from MMS to otolaryngologic management and adjuvant radiotherapy. These findings underscore the importance of multidisciplinary care in high-risk disease.

## Introduction

Epidermal inclusion cysts (EICs) are common benign lesions of the skin. Malignant transformation is rare and has been reported to occur in only 0.011-0.045% of cysts [1,2]. When present, squamous cell carcinoma (SCC) may occur within or adjacent to an EIC, typically occurring on the head and neck [1-3]. We report a case of a poorly differentiated (PD) SCC with extensive perineural invasion (PNI) and intraneural invasion (INI) that arose in an EIC on the left eyebrow.

## Case presentation

Given the clinical impression of a cyst, the lesion was clinically observed without excision or other local procedural intervention. However, persistent symptoms, including intermittent bleeding, pain, and clear drainage, raised concern for malignancy and led to a repeat biopsy, confirming the diagnosis of PD SCC.

Given the aggressive histology and high-risk location, Mohs micrographic surgery (MMS) was performed. Preoperative examination demonstrated an ill-defined crusted 1.6 cm plaque without palpable lymphadenopathy or facial nerve deficits. During MMS, serial intraoperative frozen sections were evaluated with hematoxylin and eosin (H&E) staining and revealed PD SCC with PNI. Following the seven stages of MMS, there was no definitive tumor, but dense perineural inflammation was seen in the margins, limiting assessment for residual malignancy. Therefore, the decision was made to excise additional margins for formalin-fixed paraffin-embedded (FFPE) en face sections, which revealed residual neural invasion in the margins. Immunohistochemistry was performed on the permanent sections using AE1/AE3 (pancytokeratin), p40, and S-100. AE1/AE3 and p40 confirmed infiltrative poorly differentiated squamous cell carcinoma, while S-100 outlined the involved nerve and facilitated assessment of perineural and intraneural invasion. Given the large size of the defect (11 x 7 cm), the challenges with frozen section histology, and in consideration of patient comfort and optimal management of this high-risk SCC, the patient was referred to otolaryngology for further management.

**Figure 1:**
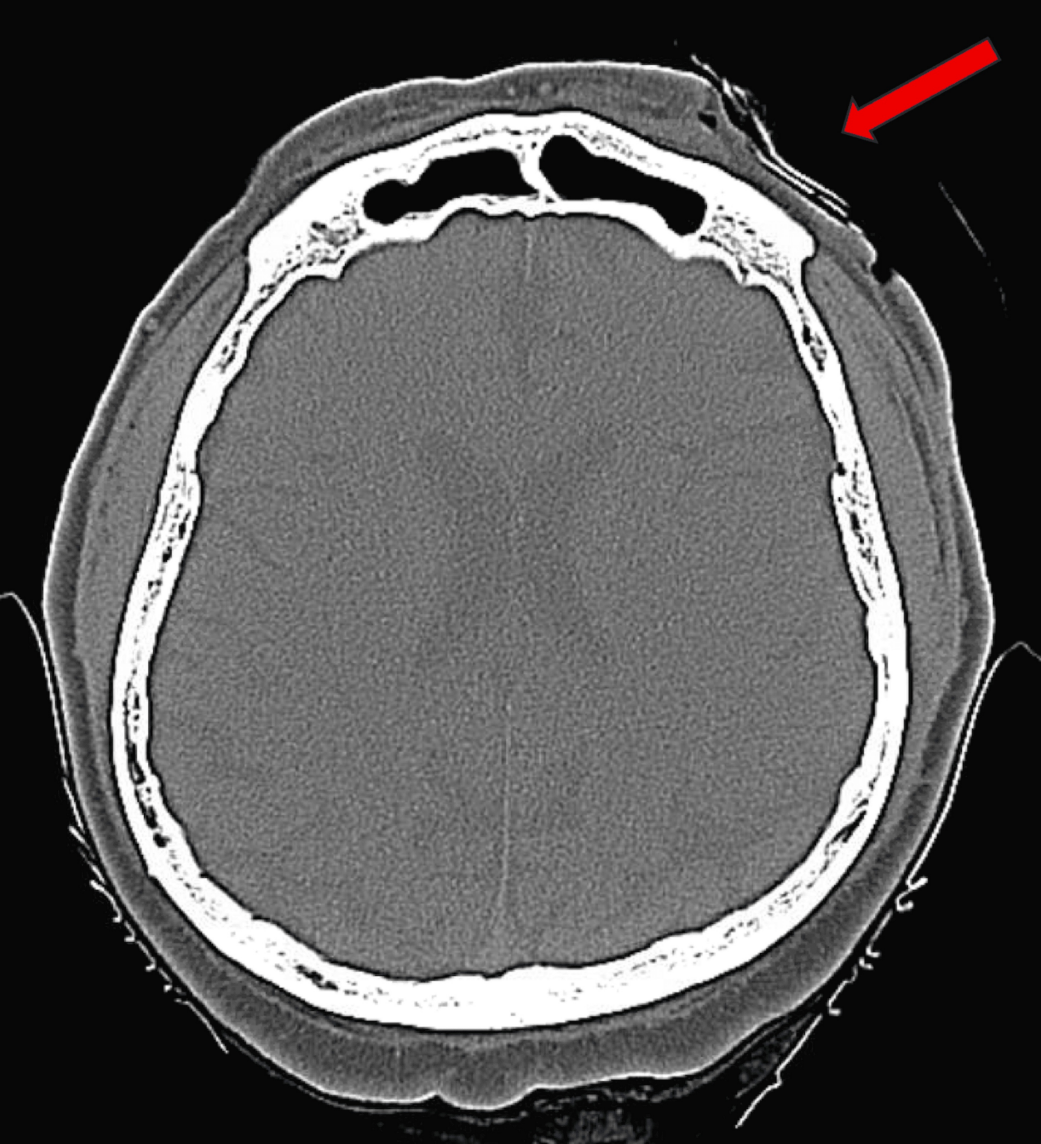
Axial computed tomography in the bone window at the level of the left eyebrow. The region of interest (red arrow) shows the intact bone and normal soft tissue without evidence of a residual tumor.

Preoperative computed tomography (CT) imaging was negative for residual tumor or lymphadenopathy (Figure 1). He subsequently underwent a left parotidectomy and selective neck dissections (levels 2A, 2B, and 3) as part of regional nodal evaluation, and an anterolateral-thigh free-flap reconstruction. Pathology confirmed no SCC in the parotid gland, and seven parotid and 22 cervical lymph nodes were negative for malignancy on routine H&E evaluation. The patient was then treated with adjuvant radiation therapy to 60 Gy in 30 fractions (2 Gy per fraction). Interim follow-up at four months demonstrated a favorable treatment response, with surveillance maxillofacial CT imaging eight months post-operatively showing no suspicious abnormality to suggest tumor recurrence. Imaging and pathology results are summarized in Table 1.

**Table 1:** Imaging and pathology results. CT, computed tomography; H&E, hematoxylin and eosin

Diagnostic step	Specimen/context	Key findings
CT face and neck	Face and neck - Preoperative Imaging	No residual tumor or lymphadenopathy
CT maxillofacial	Maxillofacial - Surveillance; Eight months post-operatively	No new suspicious abnormality to suggest tumor recurrence
Parotidectomy pathology (H&E)	Parotid gland	No squamous cell carcinoma
Lymph node pathology (H&E)	Seven parotid and 22 cervical nodes	Negative for malignancy

**Figure 2:**
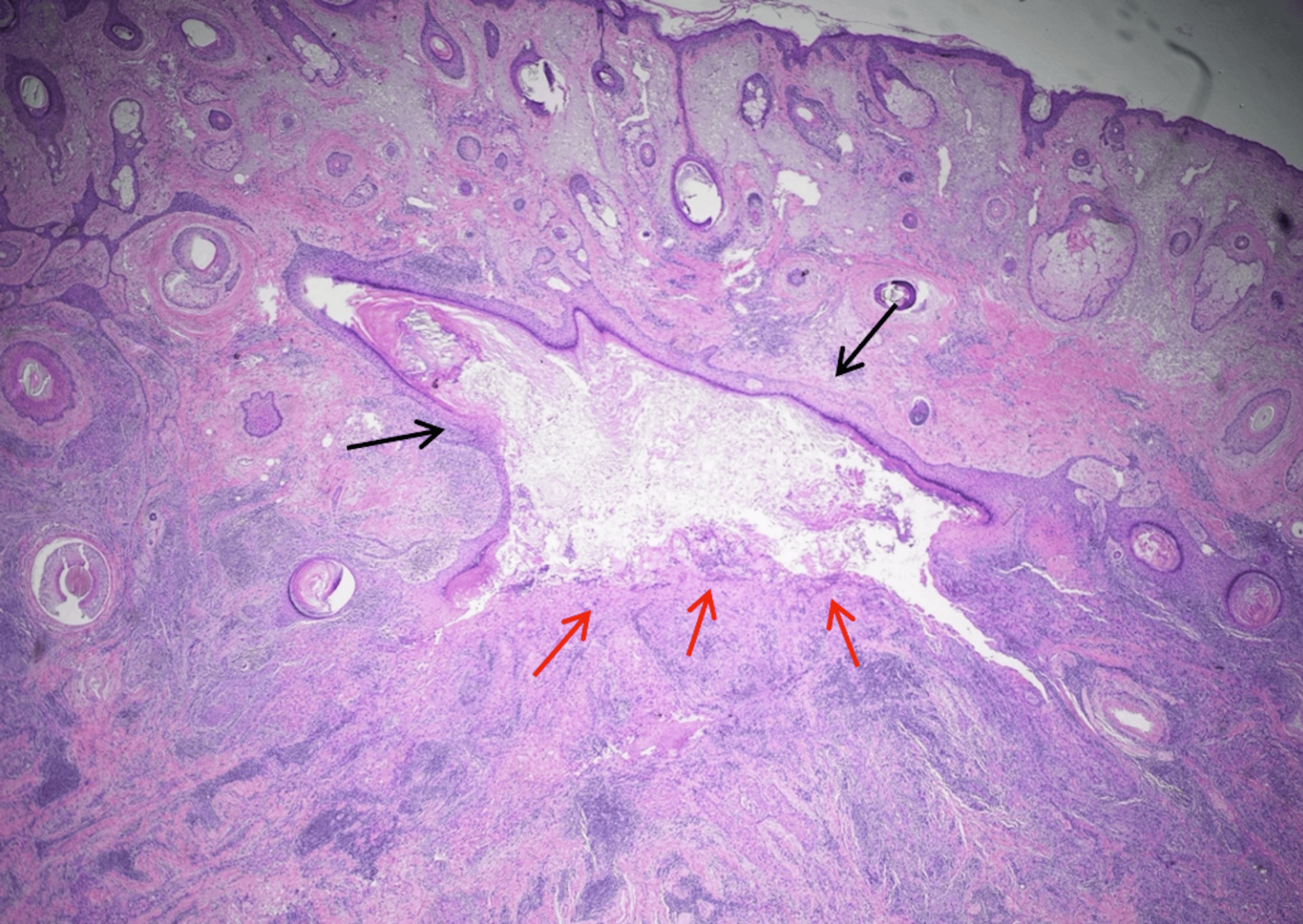
Section showing an epidermal inclusion cyst (black arrows) with infiltrative nests of a squamous cell carcinoma (red arrows) arising from the wall of the cyst. Hematoxylin and eosin stain, original magnification 2X

**Figure 3:**
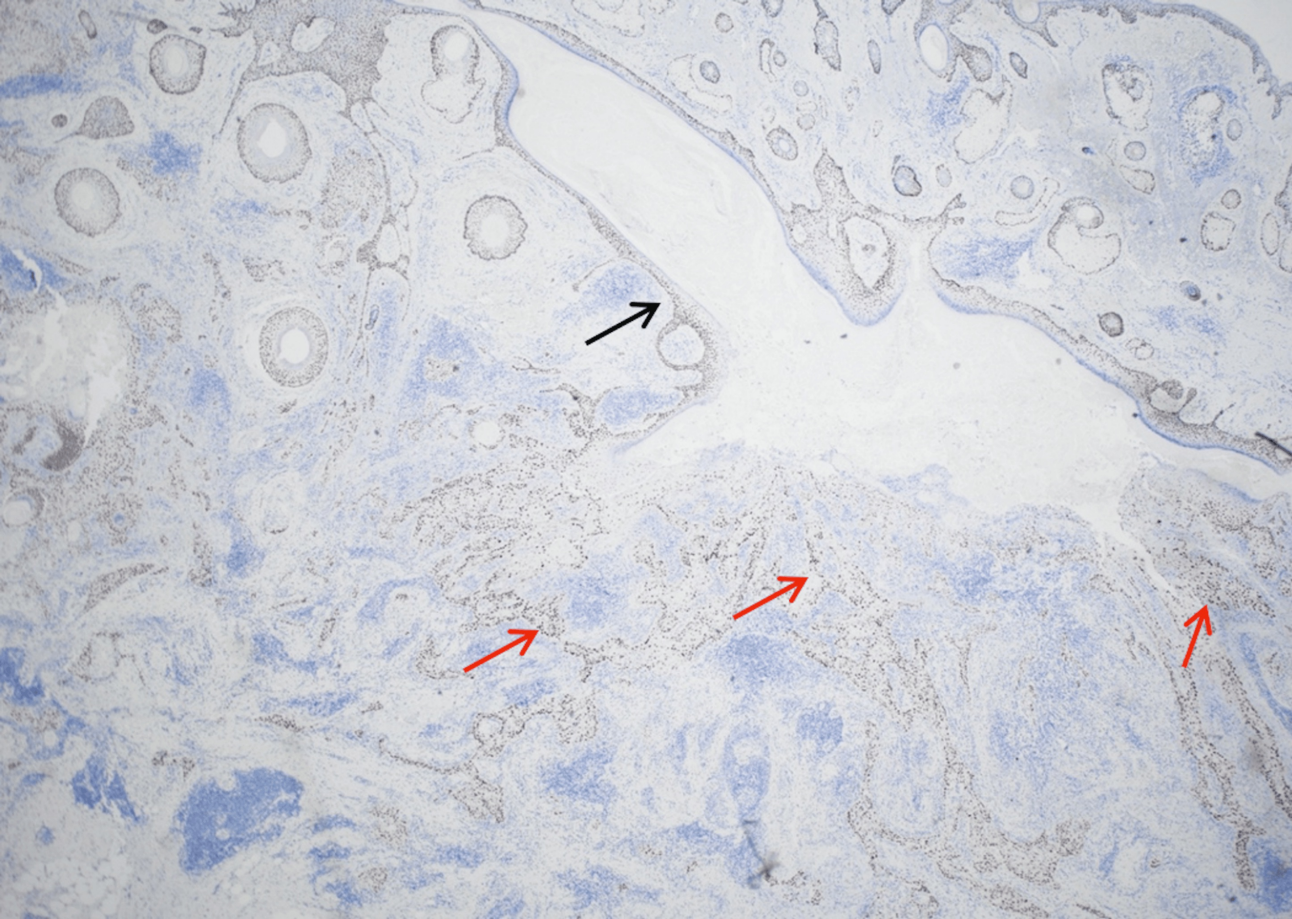
Immunostain for p40 highlighting the squamous cell carcinoma (red arrows) arising from the cyst (black arrow) and infiltrating into deep dermis and subcutaneous tissues. p40, squamous marker Immunohistochemical stain p40, original magnification 2X

**Figure 4:**
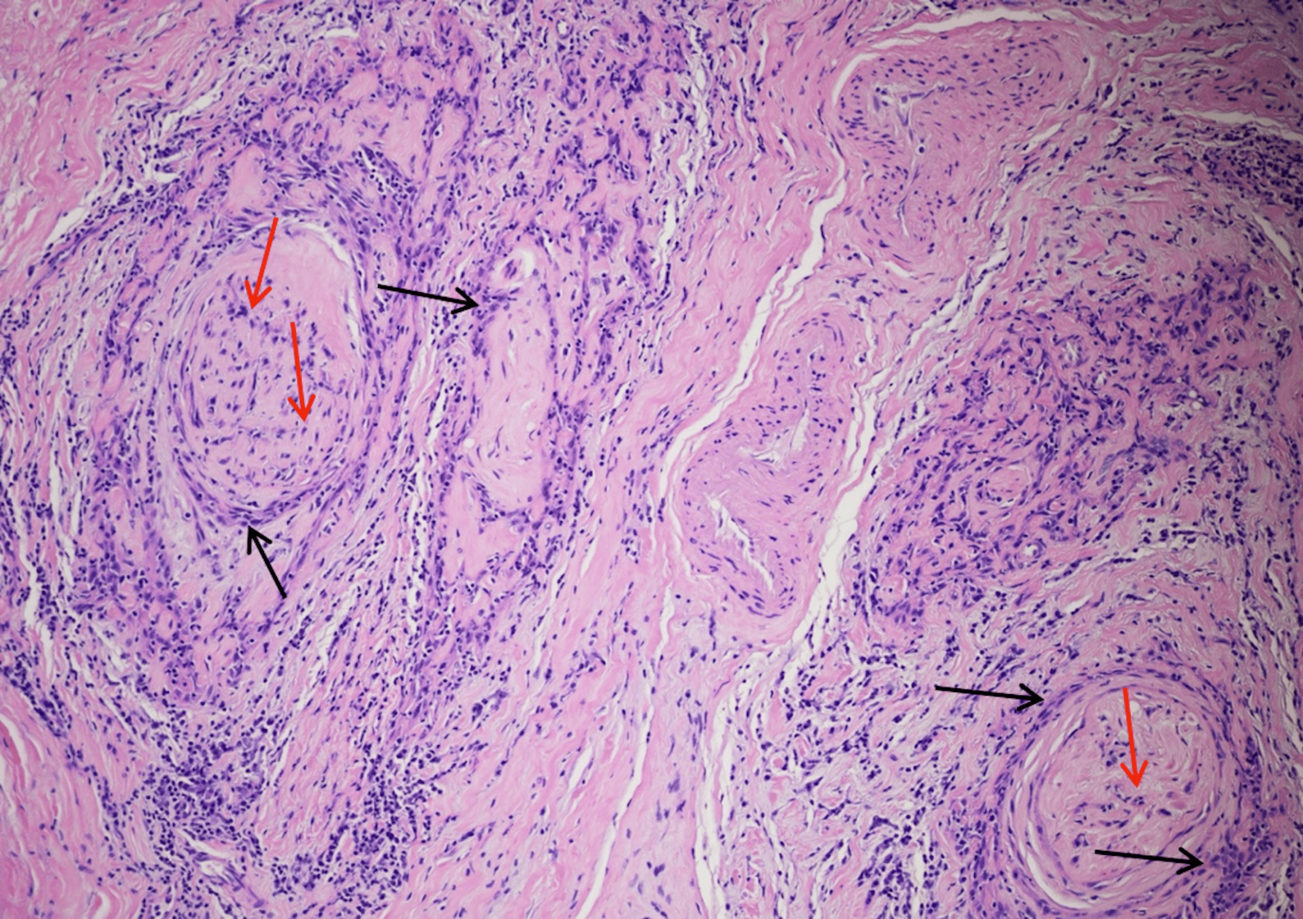
Section showing the tumor cells with extensive infiltration surrounding the nerve (black arrows) (perineural invasion) and within the nerve (red arrows) (intraneural invasion). Hematoxylin and eosin stain, original magnification 10X

**Figure 5:**
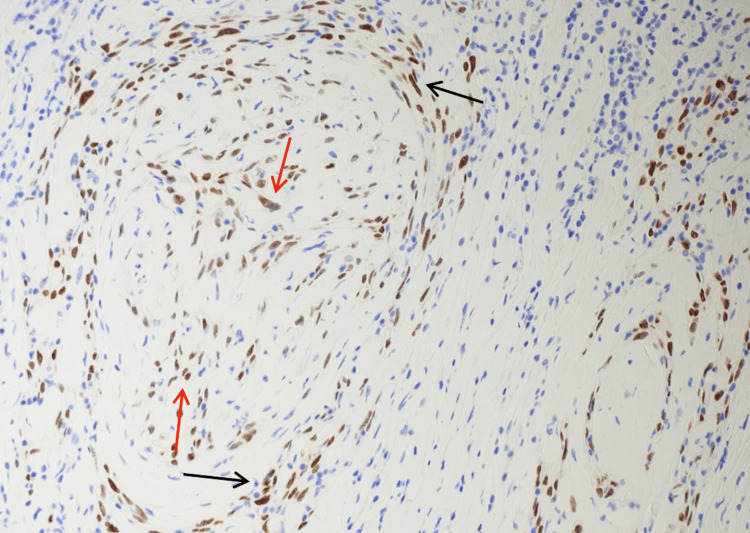
Immunostain for p40 highlighting the squamous cell carcinoma with perineural (black arrows) and intraneural invasion (red arrows). p40, squamous marker Immunohistochemical stain p40, original magnification 20X

Staging evaluation of MMS-excised tissue showed SCC arising from the wall of an EIC, with an unremarkable overlying epidermis. The bulk of the tumor was found in the deep dermis and subcutaneous tissue. H&E and p40 staining corroborated this relationship and highlighted deep dermal and subcutaneous extension (Figures 2-3). AE1/AE3 staining also supported the diagnosis of poorly differentiated SCC, and S-100 highlighted involved nerve fibers, facilitating confirmation of extensive PNI and INI. Representative H&E and p40 panels demonstrate remarkably extensive PNI and INI with involved nerves > 0.1 mm in caliber (Figures 4-5). Histopathologic findings are summarized in Table 2.

**Table 2:** Histopathology summary. SCC, squamous cell carcinoma; PNI, perineural invasion; INI, intraneural invasion; EIC, epidermal inclusion cyst; H&E, hematoxylin and eosin; FFPE, formalin-fixed paraffin-embedded; AE1/AE3, pancytokeratin; p40, squamous marker; S-100, neural marker

Diagnostic step	Specimen	Key findings and margin status
Initial biopsy	Skin, left eyebrow papule	Vascular ectasia and chronic inflammation. No cyst or malignancy identified.
Repeat biopsy	Skin, left eyebrow	Poorly differentiated cutaneous squamous cell carcinoma.
Mohs frozen sections	Serial intraoperative frozen sections from Mohs stages	Perineural inflammation limited interpretation. After seven stages, no definite tumor. Dense perineural inflammation at margins.
Permanent en face margins	Additional peripheral margins, FFPE, en face	Residual intraneural tumor at a peripheral margin involving larger caliber nerves. Margin positive for neural invasion.
Staging histology	Mohs specimen, permanent sections, FFPE	SCC arising from the wall of an epidermal inclusion cyst. Tumor centered in deep dermis and subcutis. Overlying epidermis unremarkable. Extensive perineural and intraneural invasion.
Immunohistochemistry (AE1/AE3, p40, S100)	Mohs specimen, permanent sections, immunohistochemistry	AE1/AE3 and p40 highlighted infiltrating nests of poorly differentiated squamous cell carcinoma and clarified deep extension. S-100 highlighted the involved nerve and helped delineate extensive perineural and intraneural tumor involvement.

## Discussion

Malignant transformation of an EIC to an SCC is rare, reported in 0.011-0.045% of cysts, and most often described in the head and neck, with reported lesion duration prior to SCC diagnosis ranging from months to decades [1-3]. In the largest recent case series of nine patients, risk factors included long-standing or recurrent cysts and immunosuppression, and none demonstrated PNI [2]. Our patient presented with a left eyebrow lesion that proved to be PD SCC with extensive PNI and INI, features not previously documented, to our knowledge, in SCCs arising in EICs. A targeted PubMed search using the terms “epidermal inclusion cyst”, “squamous cell carcinoma”, and “perineural” or “intraneural” revealed no previous reports of SCCs arising in an EIC with confirmed neural invasion, underscoring the unusual features of this lesion.

PNI describes tumor cells tracking along or around a nerve within its sheath, whereas INI refers to tumor infiltration of the endoneurium with partial or complete replacement of nerve fascicles. In practice, intraneural invasion is commonly reported within the broader spectrum of perineural invasion, and its independent prognostic value remains uncertain [4]. Given the prominent intraneural component in this lesion, we report INI as an explicit descriptor alongside PNI to communicate the pattern and extent of neural invasion and to inform multidisciplinary care. Permanent sections confirmed intraneural invasion within extensive neural involvement, consistent with deep local infiltration.

Both PNI and, even more rarely, INI are high-risk histopathologic features in a cutaneous SCC, associated with recurrence, metastasis, and disease-specific mortality [5,6]. PNI occurs in approximately 2-14% of cases of SCCs, whereas INI has been reported only rarely [6,7]. Notably, involvement of large-caliber nerves (≥0.1 mm) has been associated with substantially higher recurrence risk [4]. As highlighted in studies of head and neck cutaneous SCCs, such cases often require escalation beyond standard excision, with multidisciplinary input from dermatologic, surgical, and radiation oncology teams to optimize local control [4,5,7].

MMS is an effective treatment for high-risk tumors because of its high cure rates and tissue conservation. Neural invasion and PD SCC, however, may be difficult to appreciate on frozen sections. Perineural inflammation may obscure tumor involvement, yet it predicts true perineural invasion on deeper permanent levels in about half of cases, supporting correlation with permanent sections in equivocal fields [7]. As such, when histologic evaluation is limited with H&E, pairing immunohistochemical stains, such as an epithelial marker (AE1/AE3 or p40) with a neural marker (S-100 or neurofilament), helps confirm perineural tumor and clarify equivocal margins [7,8]. In our patient, the identification of extensive PNI and INI with involvement of larger-caliber nerves prompted escalation from MMS to otolaryngologic management and adjuvant radiotherapy, underscoring the importance of multidisciplinary management in high-risk disease.

## Conclusions

This report describes a case of an SCC arising in an EIC with both intraneural and extensive perineural invasion, an unusually high-risk presentation. Frozen section interpretation was limited by dense perineural inflammation, while permanent levels and selective immunohistochemistry clarified margins and guided coordinated multidisciplinary care. In our patient, the identification of extensive PNI and INI prompted escalation from MMS to otolaryngologic management and adjuvant radiotherapy, underscoring the importance of multidisciplinary management in high-risk disease.

## References

[1] Ziadi S, Trimeche M, Hammedi F, Sriha B, Jomaa W, Mokni M, Korbi S (2010). Squamous cell carcinoma arising from an epidermal inclusion cyst: a case report. N Am J Med Sci.

[2] Kim JY, Min S, Park JK, Kim SW (2024). Squamous cell carcinoma arising from epidermal cyst: a case series of 9 patients with a literature review. Ann Plast Surg.

[3] Tokunaga M, Toya M, Endo Y, Fujisawa A, Tanioka M, Kato M, Miyachi Y (2013). A case of an undifferentiated squamous cell carcinoma arising from an epidermal cyst. Case Rep Dermatol Med.

[4] Nozzoli F, Nassini R, De Logu F, Catalano M, Roviello G, Massi D (2024). Reconceiving perineural invasion in cutaneous squamous cell carcinoma: from biological to histopathological assessment. Pathobiology.

[5] Pei M, Wiefels M, Harris D (2024). Perineural invasion in head and neck cutaneous squamous cell carcinoma. Cancers (Basel).

[6] Murphy C, Zhao G, Berg D, Olson J, Argenyi Z (2015). Squamous cell carcinoma with perineural and intraneural invasion associated with hyalinized tumor nodules: a diagnostic pitfall and review of the literature. Am J Dermatopathol.

[7] Green JS, Tournas JA, Allen EJ, Youker SR, Fosko SW (2012). Mohs frozen tissue sections in comparison to similar paraffin-embedded tissue sections in identifying perineural tumor invasion in cutaneous squamous cell carcinoma. J Am Acad Dermatol.

[8] Berlingeri-Ramos AC, Detweiler CJ, Wagner RF Jr, Kelly BC (2015). Dual S-100-AE1/3 immunohistochemistry to detect perineural invasion in nonmelanoma skin cancers. J Skin Cancer.

